# Causal relationship between ankylosing spondylitis and ocular inflammatory diseases: a Mendelian randomization study

**DOI:** 10.3389/fgene.2024.1372196

**Published:** 2024-10-17

**Authors:** Yuxuan Wang, Caishun Zhang, Qing Zhang, Yutong Jiang, Yuxuan Zhang, Jing Dong

**Affiliations:** ^1^ Clinical Medicine Department, College of Basic Medicine, Qingdao University, Qingdao, China; ^2^ School of Nursing, Qingdao University, Qingdao, China; ^3^ Special Medicine Department, College of Basic Medicine, Qingdao University, Qingdao, China; ^4^ Physiology Department, College of Basic Medicine, Qingdao University, Qingdao, China

**Keywords:** ankylosing spondylitis, inflammatory diseases, keratitis, Mendelian randomization, optic neuritis, scleritis, uveitis

## Abstract

**Background:**

Observational studies have shown an increased risk of ocular inflammatory diseases in patients with ankylosing spondylitis (AS), but the genetically predicted association remains unclear. The aim of this study was to systematically assess the causal relationship between AS and ocular inflammatory diseases.

**Methods:**

We conducted a two-sample Mendelian randomization (MR) analysis to investigate the causal relationship between AS and several common ocular inflammatory diseases based on genome-wide association study (GWAS) data and public health data. Five methods, namely, inverse-variance weighted (IVW), MR–Egger, weighted median, weighted mode, and simple mode, were used. Sensitivity analysis was performed using MR–Egger intercept, Mendelian Randomization Pleiotropy RESidual Sum and Outlier (MR-PRESSO), Cochran’s Q test, outlier methods, leave-one-out analysis, and funnel plots.

**Results:**

The MR analysis showed a significantly increased risk of uveitis (OR_IVW_ = 2.825, 95%CI_IVW_ = 1.709–4.672, and P_IVW_ < 0.001), iridocyclitis (OR_IVW_ = 3.806, 95%CI_IVW_ = 2.809–5.157, and P_IVW_ < 0.001), scleritis (OR_IVW_ = 1.738, 95%CI_IVW_ = 1.190–2.539, and P_IVW_ < 0.001), and episcleritis (OR_IVW_ = 5.113, 95%CI_IVW_ = 2.067–12.645, and P_IVW_ = 0.004) associated with AS. However, no correlation was found between genetically predicted AS and keratitis (OR_IVW_ = 1.041, 95%CI_IVW_ = 0.886–1.222, and P_IVW_ = 0.628) and optic neuritis (OR_IVW_ = 0.868, 95%CI_IVW_ = 0.441–1.709, and P_IVW_ = 0.682).

**Conclusion:**

AS increases the genetically predicted risk for uveitis, iridocyclitis, scleritis, and episcleritis. No potential association of AS with keratitis and optic neuritis was found. It may provide clues for the prevention of AS complications.

## 1 Introduction

Ankylosing spondylitis (AS) is a chronic inflammatory disorder and a main outcome of rheumatic diseases for which the pathogenesis is not understood clearly (Mauro et al., 2021; Braun and Sieper, 2007). The primary pathophysiology in AS occurs in the subchondral bone and entheses (capsules, ligaments, and tendons) (Schett et al., 2017; Watad et al., 2018; Sieper et al., 2002). Intra-articular signals trigger secondary lesions of synovitis (Watad et al., 2018). Characteristic symptoms of ankylosing spondylitis are loss of spinal mobility and spinal stiffness that can be explained by structural damage and spinal inflammation (Wanders et al., 2005). Another common symptom of AS is chronic back pain that lasts a few months and often accompanied by morning stiffness. Symptoms in patients with AS have an insidious onset and inflammatory characteristics, which usually worsen at rest, improve with activity, are worse at night and in the morning, and can be improved by taking non-steroidal anti-inflammatory drugs (NSAIDs) (Navarro-Compán et al., 2021). A systematic review based on 36 studies showed that the mean prevalence of AS was 23.8 per 10,000 individuals in Europe, 31.9 in North America, 10.2 in Latin America, 16.7 in Asia, and 7.4 in Africa (Dean et al., 2014). Previous studies have shown that AS is more common in young people between 20 and 30 years old, and males are affected approximately twice more than females (Feldtkeller et al., 2003).

Extra-articular manifestations that involve multiple organs and systems are common in patients with AS and vary in incidence and severity (El, 2011). As early as the 1970s, a potential association between AS and several other diseases, such as inflammatory bowel disease (IBD) and psoriasis, was observed (Moll et al., 1974). Uveitis is the most common extra-articular manifestation of AS, with a prevalence of 20%–30% during the disease course, which increases with its duration. In these cases, acute anterior uveitis accounts for 90% (Zeboulon et al., 2008). The involvement of the gastrointestinal tract causes intestinal tissue inflammation and inflammatory bowel disease (De Keyser and Mielants, 2003; Rudwaleit and Baeten, 2006). Bone tissue involvement can increase the risk of osteoporosis and vertebral fractures (Ghozlani et al., 2009; Ulu et al., 2014). Skin involvement may result in psoriasis (Machado et al., 2013), while heart involvement can lead to ischemic heart disease (IHD) (Essers et al., 2016). These complications significantly influence the survival rate of patients with AS.

We noted that some studies have reported cases of AS complicated with other ocular inflammation conditions, including scleritis, keratitis, optic neuritis, and iridocyclitis (Karia et al., 1999; Díaz-Valle et al., 2009; Yoshikawa et al., 1992; Zhao et al., 2015; Lee et al., 2022). This suggests that there may be a potential association between ankylosing spondylitis and ocular inflammation.

Traditional observational studies are susceptible to confounding factors in the exposure environment and prone to reverse causality. Therefore, whether AS is causally linked to ocular inflammatory diseases remains undetermined. Establishing the potential links between AS and ocular inflammatory diseases is crucial for enhancing the prevention of AS complications.

Mendelian randomization (MR) is a statistical approach based on genome-wide association studies (GWASs) used to construct instrumental variables (IVs). When the basic assumptions are met, it can effectively reduce confounding factors and reverse causality to determine the association between exposure and outcome (Davies et al., 2018; O’Donnell and Sabatine, 2018). In this study, we conducted a two-sample MR analysis to explore the causal relationship between AS and ocular inflammatory diseases.

## 2 Materials and methods

### 2.1 Study design

**FIGURE 1:**
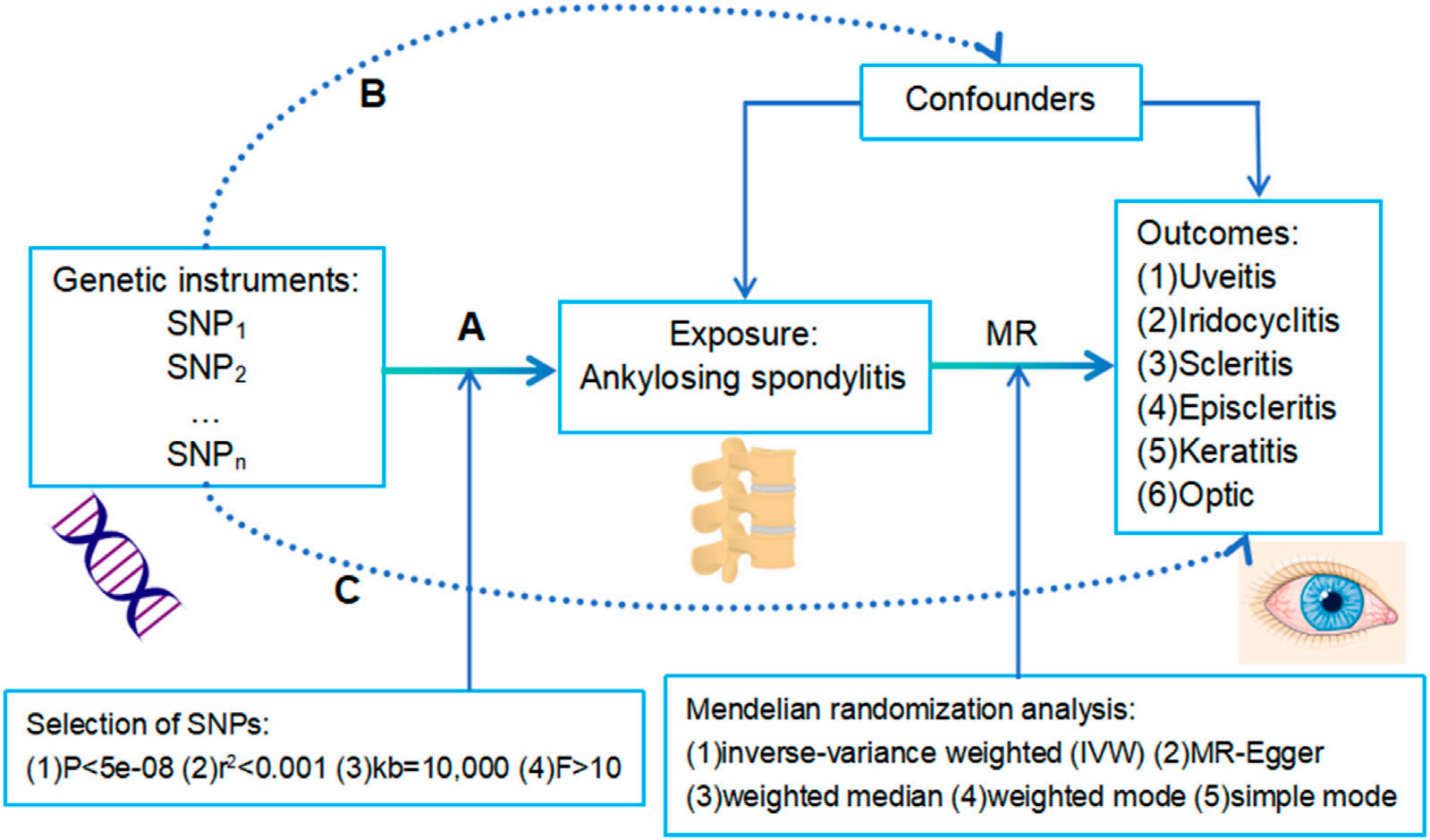
Overview of this study and the three core hypotheses **(A–C)** of MR design. **(A)** The genetic instruments proposed as instrumental variables should be strongly associated with exposure (AS). Selection criteria included 1) *p* < 5e-08; 2) r^2^ < 0.001; 3) kb = 10,000; and 4) F > 10. **(B)** The instrumental variables should be independent of other confounding factors that may influence the association between exposure and outcome. **(C)** The instrumental variables were not directly related to the outcomes (uveitis, scleritis, keratitis, optic neuritis, iridocyclitis, and episcleritis).

An overview of this study and the three core hypotheses of MR are shown in Figure 1. The genetic instruments included as IVs in the MR study should satisfy the three basic hypotheses: A) the genetic IVs should be strongly associated with exposure (AS); B) the genetic IVs should be independent of other confounding factors that may influence the association between exposure and outcomes (uveitis, iridocyclitis, scleritis, episcleritis, keratitis, and optic neuritis); and C) the genetic IVs should not be directly related to the outcomes. We performed multiple methods of testing for horizontal pleiotropy to test the second and third hypotheses.

### 2.2 Data sources

GWAS data for MR were all derived from populations of European ancestry. Table 1 provides detailed information about the data sources and the demographic profiles of AS, uveitis, scleritis, keratitis, optic neuritis, iridocyclitis, and episcleritis. GWAS data associated with AS were collected from the International Genetics of Ankylosing Spondylitis (IGAS) Consortium comprising 22,647 subjects (9,069 cases and 13,578 controls) (Cortes et al., 2013). GWAS data associated with uveitis were collected from a GWAS meta-analysis study that included 480,742 subjects (2,616 cases and 478,126 controls) (Sakaue et al., 2021). Other outcome GWAS data, including on keratitis, scleritis, episcleritis, iridocyclitis, and optic neuritis, were derived from the FinnGen R10 database (https://www.finngen.fi) (Kurki et al., 2023). The FinnGen database combined genotype data generated from legacy samples from Finnish biobanks and digital health record data from Finnish health registries. It aimed to provide a new insight into disease genetics. The data used in our study were all previously published and openly accessible to researchers, so no additional ethical approval was required.

**TABLE 1:** Data sources and demographic profiles in this study.

Exposure or outcome	Sample size (cases/controls)	Ancestry	Consortia	PubMed id or url
AS	9,069/13,578	European	IGAS Consortium	23749187
Uveitis	2,616/478,126	European	GWAS meta-analysis study	34594039
Keratitis	12,916/390,647	European	FinnGen consortium	(https://www.finngen.fi)
Scleritis	327/390,647	European	FinnGen consortium	(https://www.finngen.fi)
Episcleritis	1,546/390,647	European	FinnGen consortium	(https://www.finngen.fi)
Iridocyclitis	8,016/390,647	European	FinnGen consortium	(https://www.finngen.fi)
Optic neuritis	1,295/409,190	European	FinnGen consortium	(https://www.finngen.fi)

### 2.3 Selection of instrumental variables

In our MR analysis, we applied consistent standard screening and a series of quality-control procedures to obtain IVs that met three core assumptions.

The significance criterion (*p* < 5e–08) was set to select genetic instruments for AS in association studies. Then, the clumping procedure was conducted with r^2^ < 0.001 and a window size of 10,000 kb based on the data from the 1000 Genomes Project in individuals of European ancestry (Abecasis et al., 2010). The proxy SNP from the 1000 Genomes Project was used as the surrogate effect allele when a requested SNP was absent. MR-Base provided LD proxy lookups in this case (Hemani et al., 2018). Then, we excluded palindromic SNPs and SNPs with non-concordant alleles by harmonizing the exposure and outcome data. Finally, we obtained a summary of SNPs with same-effect alleles for both exposure and outcome. Furthermore, the F-statistic was obtained from each included SNP to remove SNPs that were weakly associated with exposure (F < 10) (Hemani et al., 2018). The F-statistic was calculated using the following formula: F = R^2^ × (N-2)/(1-R^2^), where R^2^ = β^2^_exposure_/(β^2^_exposure_ + SE^2^_exposure_ × N). N represents the sample size of the GWAS, SE represents the standard error, and beta represents the estimated genetic effect. There were 25 SNPs conducted into the final set (Supplementary Table S1).

### 2.4 Statistical analysis

We used inverse-variance weighted (IVW) as the primary analysis method to test for causality between exposures and outcomes, which can return the unbiased estimates (Burgess et al., 2015). In addition, MR–Egger, weighted median, weighted mode, and simple mode methods were used to estimate the effect of exposure on outcomes (Bowden et al., 2015; Bowden et al., 2016; Hartwig et al., 2017). If the effects estimated by these methods were consistent with the IVW, the analysis results can be considered reliable.

We performed MR analysis in R (version 4.3.1) with the R packages “TwoSampleMR” (version 0.5.8) and “MRPRESSO” (Hartwig et al., 2017).

### 2.5 Sensitivity analysis

The heterogeneity was tested using the Cochran’s Q statistic, and the leave-one-out test was used to determine whether a single SNP drove the overall effect (Burgess et al., 2013). We conducted the Mendelian Randomization Pleiotropy RESidual Sum and Outlier (MR-PRESSO) test and MR–Egger regression to assess horizontal pleiotropy (Bowden et al., 2015; Hartwig et al., 2017). The mean pleiotropic effect of IVs was represented by the intercept term of MR–Egger regression. We removed outliers with a *p*-value of less than 0.05 that had been detected by the MR-PRESSO global test and reanalyzed the data to obtain more accurate results. Finally, we examined the relationship between the selected SNPs and any possible confounding factors by searching the PhenoScannerV2 website, PhenoScanner (cam.ac.uk).

## 3 Results

The quality control and screening procedures described above showed that the number of IVs included in the analysis to explore the effect of AS on various ocular inflammations varied from 20 to 24. Each independent IV included in the analysis had an F-value greater than 10, indicating an absence of bias resulting from weak IVs. Supplementary Table S1 provides detailed information.

**FIGURE 2:**
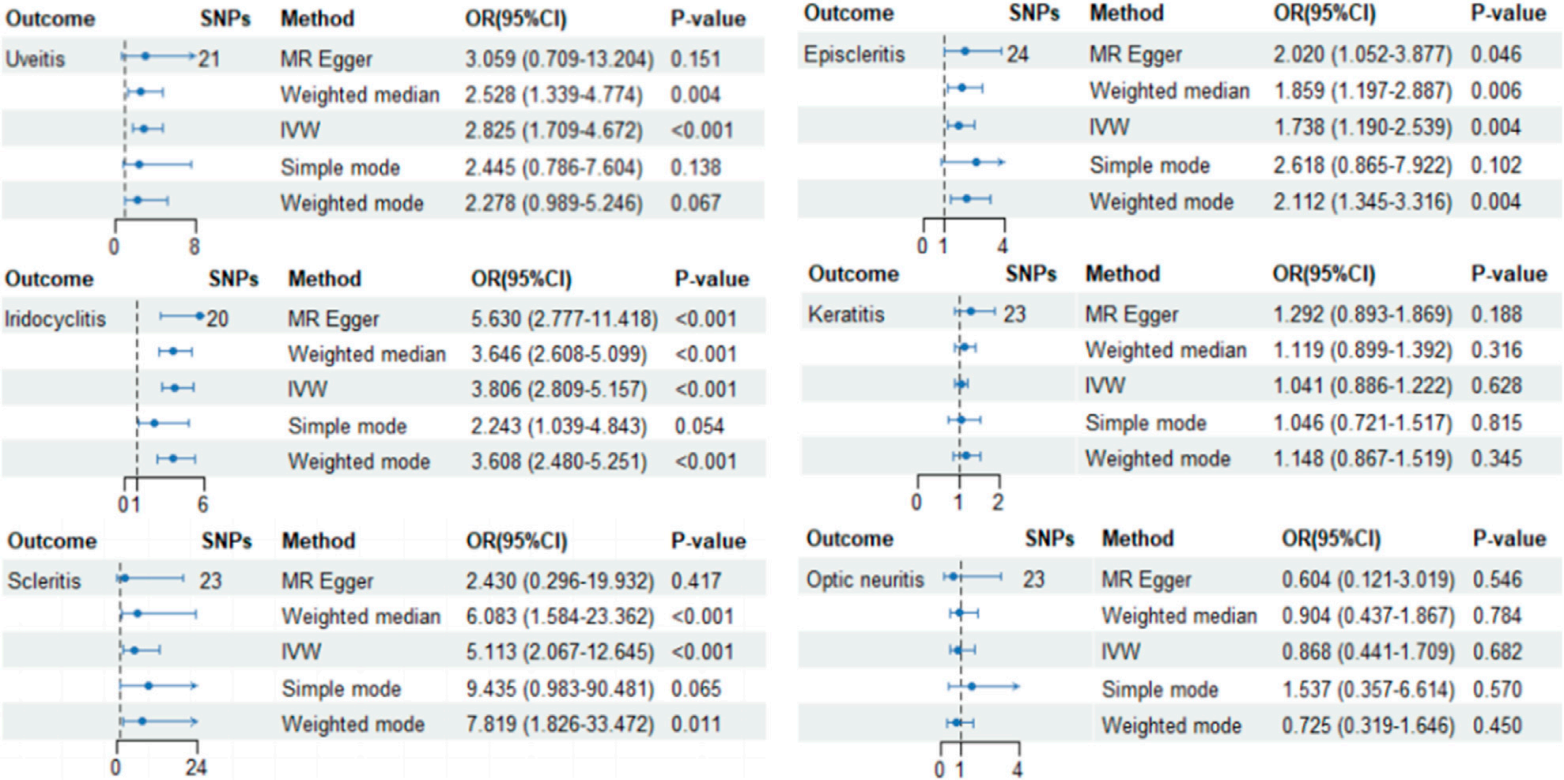
Causal effects of AS on ocular inflammatory diseases (uveitis, iridocyclitis, scleritis, episcleritis, keratitis, and optic neuritis) using different MR methods. The blue dot in the figure represents the OR value, and the blue line segment represents 95%CI.

**FIGURE 3:**
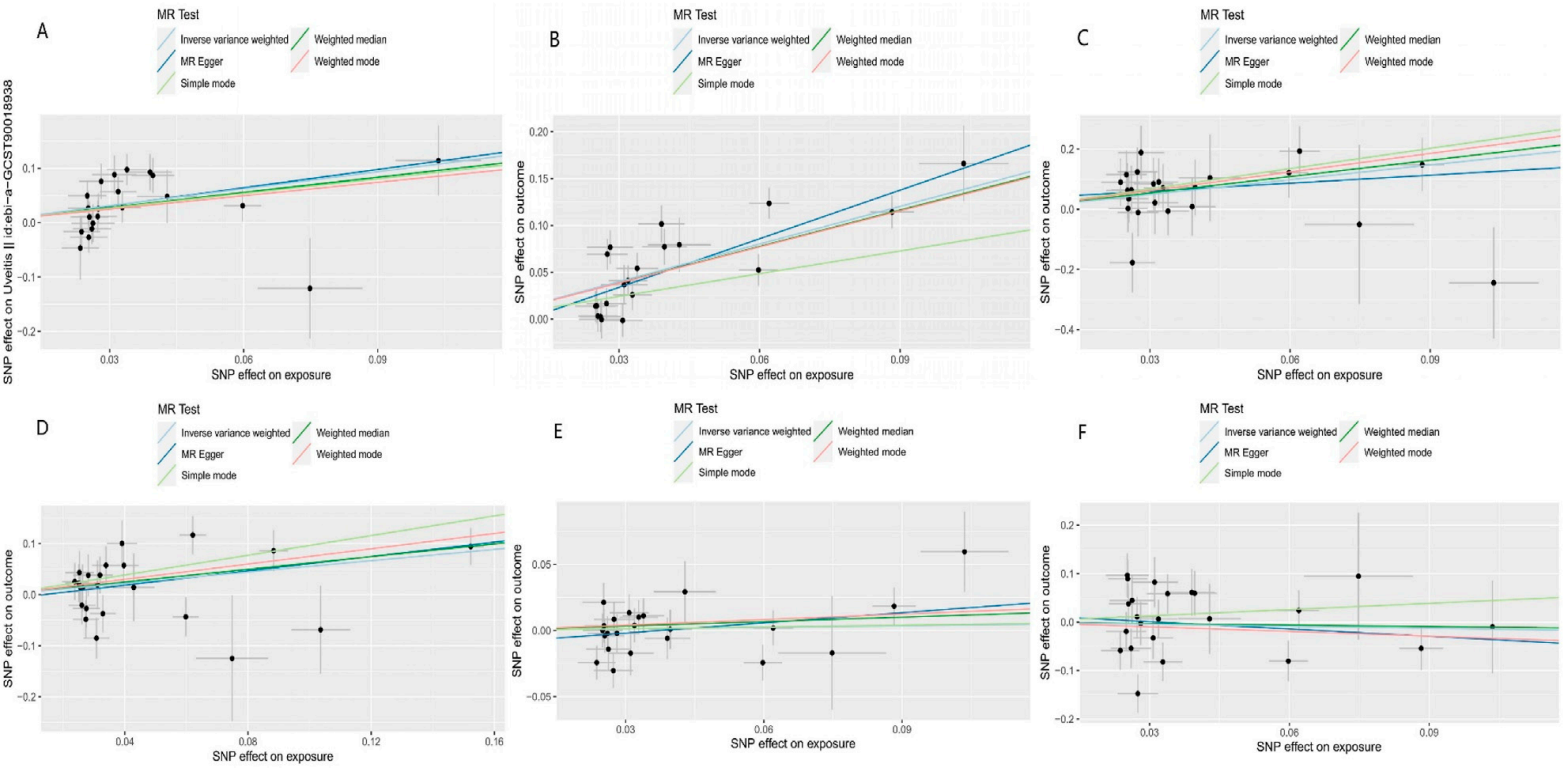
Scatter plot of the causal relationships between AS and outcomes using different methods: **(A)** causal estimates for AS on uveitis; **(B)** causal estimates for AS on iridocyclitis; **(C)** causal estimates for AS on scleritis; **(D)** causal estimates for AS on episcleritis; **(E)** causal estimates for AS on keratitis; and **(F)** causal estimates for AS on optic neuritis. Each black dot in this figure indicates an SNP, and the lines correspond to the effects of AS on ocular inflammatory diseases using different MR methods.

Our results suggest that AS is associated with an increased risk of uveitis (OR_IVW_ = 2.825, 95%CI_IVW_ = 1.709–4.672, and P_IVW_ < 0.001), iridocyclitis (OR_IVW_ = 3.806, 95%CI_IVW_ = 2.809–5.157, and P_IVW_ < 0.001), scleritis (OR_IVW_ = 1.738, 95%CI_IVW_ = 1.190–2.539, and P_IVW_ < 0.001), and episcleritis (OR_IVW_ = 5.113, 95%CI_IVW_ = 2.067–12.645, and P_IVW_ = 0.004) in the European population. Figure 2 shows the results of the association between AS and ocular inflammatory diseases using IVW, MR–Egger, weighted median, simple mode, and weighted mode methods. The blue dot in the figure represents the OR value, and the blue line segment represents 95%CI. The scatter plot shows the causal relationships between AS and ocular inflammatory diseases using different methods (Figure 3). Each black dot in this figure indicates an SNP, and the lines correspond to the effects of AS on ocular inflammatory diseases using different MR methods.

**FIGURE 4:**
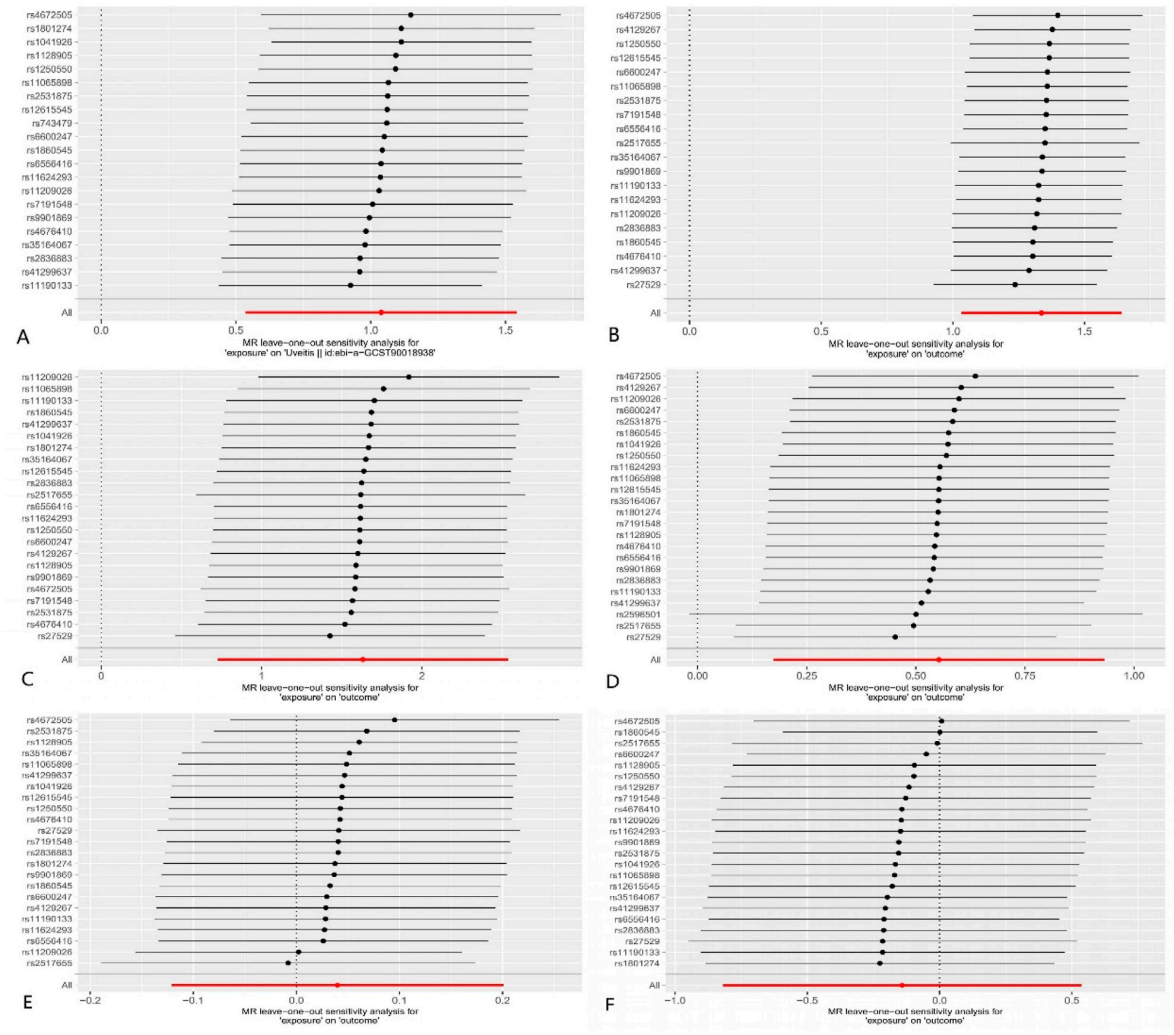
MR leave-one-out analysis: **(A)** AS on uveitis; **(B)** AS on iridocyclitis; **(C)** AS on scleritis; **(D)** AS on episcleritis; **(E)** AS on keratitis; and **(F)** AS on optic neuritis. Dots and lines in this figure represent the effect of an SNP.

The results of the MR–Egger intercept indicated that there was no horizontal pleiotropy in the four groups (*p* > 0.05). The leave-one-out test showed that the overall effect was not driven by the individual SNP (Figure 4). The heterogeneity test showed no heterogeneity between SNPs in the MR analysis of AS on scleritis (Q_MR–Egger_ = 18.421, *p* = 0.622; Q_IVW_ = 19.010, *p* = 0.645) and episcleritis (Q_MR–Egger_ = 33.767, *p* = 0.051; Q_IVW_ = 34.246, *p* = 0.062). However, the heterogeneity test showed heterogeneity between SNPs in the MR analysis of AS on uveitis (Q_MR–Egger_ = 31.730, *p* = 0.034; Q_IVW_ = 31.751, *p* = 0.046) and iridocyclitis (Q_MR–Egger_ = 44.674, *p* < 0.001; Q_IVW_ = 48.244, *p* < 0.001). We used the random-effects model as a random-effects IVW approach allowed the existence of heterogeneity. Supplementary Figure S1 shows the funnel plots.

No correlation was found between genetically predicted AS and keratitis (OR_IVW_ = 1.041, 95%CI_IVW_ = 0.886–1.222, and P_IVW_ = 0.628) and optic neuritis (OR_IVW_ = 0.868, 95%CI_IVW_ = 0.441–1.709, and P_IVW_ = 0.682).

## 4 Discussion

In this study, we performed a two-sample MR to explore the relationship between AS and ocular inflammatory diseases. We found that AS increased genetically predicted risk for uveitis, iridocyclitis, scleritis, and episcleritis through large-scale GWAS data.

Previous studies on the ocular extra-articular manifestations of AS have primarily focused on the anterior uveitis, which is considered to be the most common extra-articular manifestation of AS (Braun and Sieper, 2007; Zeboulon et al., 2008). Uveitis is an inflammation of the uveal tract of the middle layer of the eye, which includes the iris, ciliary body, and choroid. Inflammation in the anterior layer of the uvea mainly affects the iris and ciliary body, known as iridocyclitis, whereas inflammation in the posterior uvea mainly affects the retina and choroid, also known as chorioretinitis (Selmi, 2014; Tuğal-Tutkun, 2023). An epidemiological study conducted in North America showed an incidence of uveitis of 52.4 per 100,000 person-years and a prevalence rate of 115.3 per 100,000 person-years (Gritz and Wong, 2004). Clinically, uveitis is often acute in onset and characterized by periorbital pain, photophobia, redness, and blurred vision. The prognosis for acute uveitis is generally positive, and satisfactory outcomes can be achieved with timely and effective treatment. However, if left undiagnosed and untreated, it can also turn into chronic progression that may result in a series of severe complications, including glaucoma, macular edema, cataract, iris, and lens adhesion, and even permanent visual loss (Selmi, 2014; McCluskey et al., 2000). Clinicians should remain vigilant for the potential development of uveitis in patients diagnosed with AS. Early diagnosis and treatment of uveitis are of great significance to improve the prognosis of patients and the medical burden on society.

The human major histocompatibility complex (MHC) comprises human leukocyte antigens (HLAs) encoded on chromosome 6 (Bowness, 2015). It was recognized that AS was significantly associated with HLA loci (HLA B27) in pathogenesis and stronger than any other rheumatic disease, as reported decades ago (Brewerton et al., 1973a; Schlosstein et al., 1973). Epidemiological studies have shown that HLA B27 is present in over 90% of patients with AS, and the development of AS carries a risk as high as approximately 5% in individuals who are HLA B27-positive (Khan, 1996; van der Linden et al., 1984). Positivity for HLA B27 can explain 20%–30% of the genetic risk (Braun and Sieper, 2007). In addition to HLA B27, HLA DR1 and HLA B60 may also be associated with AS (Rudwaleit and Höhler, 2001). Possible targets of an autoimmune response in AS are proteoglycan and proteoglycan II (McGonagle et al., 1998). Typical features of AS were presented in animals immunized with proteoglycan (Zhang, 2003).

During the same period, Brewerton et al*.* observed that around half of the patients with acute anterior uveitis were HLA B27-positive, indicating a significant association between HLA B27 and its pathogenesis. This finding established a genetic link between AS and acute anterior uveitis (Brewerton et al., 1973b). Researchers suggested that HLA B27 may mediate changes in the normal gut microbiota, causing secondary inflammation that leads to the development of AS and uveitis (Hammer et al., 1990; Rosenbaum and Asquith, 2018). In our analysis, we used two-sample MR analysis to confirm that the risk of uveitis and anterior uveitis (iridocyclitis) is significantly associated with AS. Unfortunately, in the reverse MR analysis, we tried but failed to identify sufficient IVs to demonstrate that uveitis increases the risk of AS. This may be due to the insufficient sequencing depth of GWAS data for AS that we used (total number of SNPs = 99,962).

The analysis results showed a significant increase in the genetically predicted risk of scleritis and episcleritis in patients with AS. More attention seems to have been paid to the association between rheumatoid arthritis and scleritis. However, very few studies have explored the risk of scleral inflammation (scleritis and episcleritis) in patients with AS or simply considered scleritis a potential complication of uveitis. The sclera belongs to the outer structure of the ocular wall, accounting for about five–sixth of the entire outer wall. Scleritis is a heterogeneous group of chronic autoimmune diseases centered on the sclera, involving adjacent structures including the cornea and uvea (Okhravi et al., 2005; Lin et al., 2006). Scleritis is characterized by the insidious onset of redness and ocular pain. The severity of scleritis ranges from mild self-limited inflammation in the superficial layer to severe necrosis of the underlying layer, and its severe complications include glaucoma, cataract, uveitis, and corneal perforation (Wakefield et al., 2013; Sainz de la Maza et al., 1994). In recent years, many cases of severe scleritis have been recorded (Raven et al., 2016). The pathogenesis of scleritis is still unclear. It has been found that matrix metalloproteinases (MMPs) are expressed in the sclera tissues affected by necrotizing scleritis and modulated by cytokines, so this tissue necrosis may be partly attributed to MMP activity (Wakefield et al., 2013; Di Girolamo et al., 1997). Apart from that, scleritis and episcleritis should not be confused. Episcleritis is typically characterized by redness, mild ocular discomfort, and pain. In contrast, scleritis is associated with more severe pain and tenderness that is not present in patients with episcleritis (Wakefield et al., 2013; Watson and Hayreh, 1976). Therefore, clinicians should be aware of the risk of scleritis and episcleritis in patients with AS, in addition to uveitis.

Keratitis can be caused by various factors, such as herpes simplex virus infection, fungal infection, and *Acanthamoeba* infection (Author Anonymous, 1988; Rowe et al., 2013; Brown et al., 2021). In our analysis, we did not identify a potential link between keratitis and AS. However, attention should be paid to the possibility of keratitis secondary to other ocular inflammation. Similarly, we found no potential association between AS and optic neuritis. Current studies suggest that there is a strong association between optic neuritis and multiple sclerosis (MS). About 70% of MS patients present with optic neuritis (Toosy et al., 2014).

Major strengths of this study include the following: we performed MR analysis using large-scale GWAS data, effectively eliminating possible confounding factors in observational studies and using multiple analysis methods to obtain robust results. GWAS data on AS and ocular inflammatory diseases were collected by different consortiums, so there was almost no overlap in the different groups of samples.

There are also some limitations to our study. First, all the samples were obtained from European populations, so the results cannot be effectively generalized to other races or regions. In addition, heterogeneity was identified in certain group analyses. Furthermore, we were unable to elucidate the exact mechanism of ocular inflammation caused by AS. Based on the available statistics, we failed to extract enough IVs in the reverse MR analysis to obtain the possibility of reverse causality between ocular inflammatory diseases and AS, which may be due to the insufficient sequencing depth of GWAS data for AS (total number of SNPs = 99,962). Therefore, the results of this study have diagnostic limitations. Future studies with larger sample size are needed to verify our results.

## 5 Conclusion

In summary, the evidence presented in the two-sample MR analysis suggests that there may be a causal relationship between genetically predicted AS and ocular inflammatory diseases. Our results showed that AS increased the genetically predicted risk for uveitis, iridocyclitis, scleritis, and episcleritis. Future studies are needed to elucidate the specific mechanisms. Researchers and clinicians should be aware of the risk of ocular inflammation in patients with AS.

## Data Availability

The original contributions presented in the study are included in the article/Supplementary Material; further inquiries can be directed to the corresponding author.

## Abbreviations

AS, ankylosing spondylitis; GWAS, genome-wide association study; HLA, human leukocyte antigen; IBD, inflammatory bowel disease; IGAS, International Genetics of Ankylosing Spondylitis; IHD, ischemic heart disease; IVs, instrumental variables; IVW, inverse-variance weighted; MHC, major histocompatibility complex; MMP, matrix metalloproteinase; MR, Mendelian randomization; MR-PRESSO, Mendelian Randomization Pleiotropy RESidual Sum and Outlier; MS, multiple sclerosis; NSAIDs, non-steroidal anti-inflammatory drugs.
