# Supplementary material for: Causal relationship between ankylosing spondylitis and ocular inflammatory diseases: a Mendelian randomization study
Source: Front Genet. 2024 Oct 17;15:1372196. doi: 10.3389/fgene.2024.1372196 (PMC11524906; doi:10.3389/fgene.2024.1372196)
Supplement: Supplementary file 1 [file Table2.DOCX]

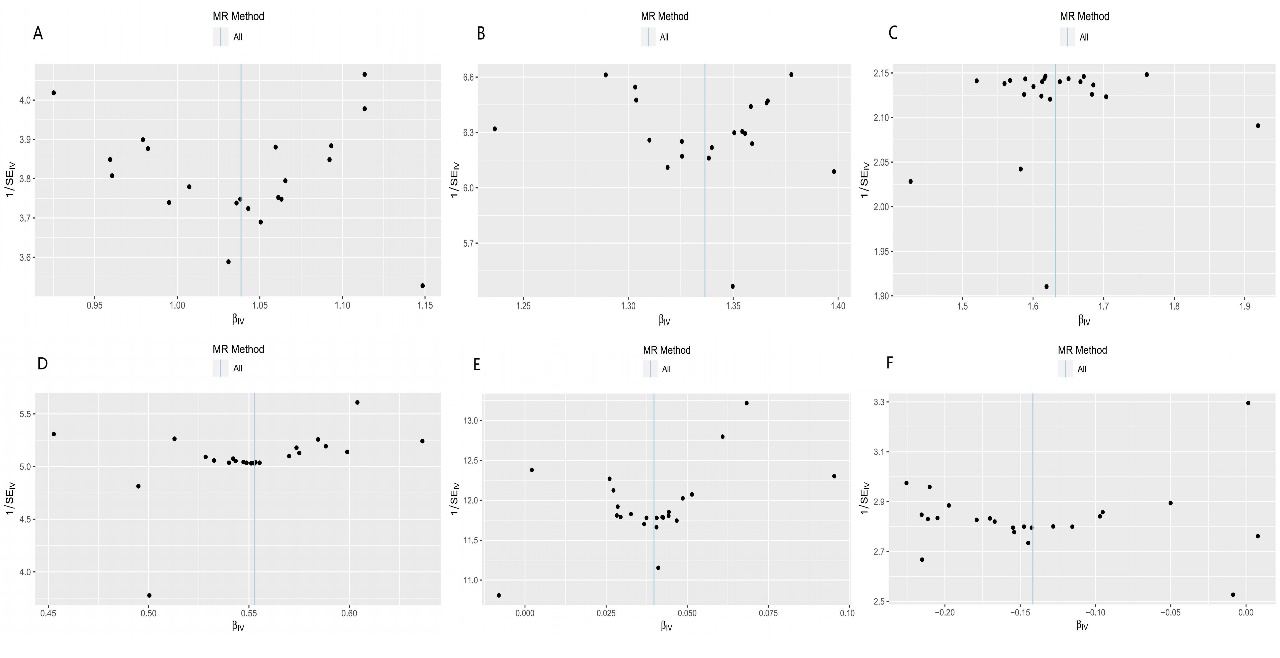


Supplement Figure: Funnel plot (A: AS on uveitis; B: AS on iridocyclitis; C: AS on scleritis; D: AS on episcleritis; E: AS on keratitis; F: AS on optic neuritis).
